# Effect of functional sympathetic nervous system impairment of the liver and abdominal visceral adipose tissue on circulating triglyceride-rich lipoproteins

**DOI:** 10.1371/journal.pone.0173934

**Published:** 2017-03-27

**Authors:** Michael F. La Fountaine, Christopher M. Cirnigliaro, Steven C. Kirshblum, Cristin McKenna, William A. Bauman

**Affiliations:** 1 Department of Veterans Affairs Rehabilitation Research & Development Service National Center for the Medical Consequences of Spinal Cord Injury, James J. Peters Veterans Affairs Medical Center, Bronx, New York, United States of America; 2 Department of Medicine, Icahn School of Medicine at Mount Sinai, New York, New York, United States of America; 3 School of Health and Medical Sciences, Seton Hall University, South Orange, New Jersey, United States of America; 4 The Institute for Advanced Study of Rehabilitation and Sports Science, School of Health and Medical Sciences, Seton Hall University, South Orange, New Jersey, United States of America; 5 Kessler Institute for Rehabilitation, West Orange, New Jersey, United States of America; 6 Department of Physical Medicine and Rehabilitation, Rutgers New Jersey Medical School, Newark, New Jersey, United States of America; 7 Department of Rehabilitation Medicine, Icahn School of Medicine at Mount Sinai, New York, New York, United States of America; East Tennessee State University, UNITED STATES

## Abstract

**Background:**

Interruption of sympathetic innervation to the liver and visceral adipose tissue (VAT) in animal models has been reported to reduce VAT lipolysis and hepatic secretion of very low density lipoprotein (VLDL) and concentrations of triglyceride-rich lipoprotein particles. Whether functional impairment of sympathetic nervous system (SNS) innervation to tissues of the abdominal cavity reduce circulating concentrations of triglyceride (TG) and VLDL particles (VLDL-P) was tested in men with spinal cord injury (SCI).

**Methods:**

One hundred-three non-ambulatory men with SCI [55 subjects with neurologic injury at or proximal to the 4^th^ thoracic vertebrae (↑T4); 48 subjects with SCI at or distal to the 5^th^ thoracic vertebrae (↓T5)] and 53 able-bodied (AB) subjects were studied. Fasting blood samples were obtained for determination of TG, VLDL-P concentration by NMR spectroscopy, serum glucose by autoanalyzer, and plasma insulin by radioimmunoassay. VAT volume was determined by dual energy x-ray absorptiometry imaging with calculation by a validated proprietary software package.

**Results:**

Significant group main effects for TG and VLDL-P were present; post-hoc tests revealed that serum TG concentrations were significantly higher in ↓T5 group compared to AB and ↑T4 groups [150±9 vs. 101±8 (p<0.01) and 112±8 mg/dl (p<0.05), respectively]. VLDL-P concentration was significantly elevated in ↓T5 group compared to AB and ↑T4 groups [74±4 vs. 58±4 (p<0.05) and 55±4 μmol/l (p<0.05)]. VAT volume was significantly higher in both SCI groups than in the AB group, and HOMA-IR was higher and approached significance in the SCI groups compared to the AB group. A linear relationship between triglyceride rich lipoproteins (i.e., TG or Large VLDL-P) and VAT volume or HOMA-IR was significant only in the ↓T5 group.

**Conclusions:**

Despite a similar VAT volume and insulin resistance in both SCI groups, the ↓T5 group had significantly higher serum TG and VLDL-P values than that observed in the ↑T4 and the AB control groups. Thus, level of injury is an important determinate of the concentration of circulating triglyceride rich lipoproteins, which may play a role in the genesis of cardiometabolic dysfunction.

## Background

Autonomic nervous system (ANS) dysfunction contributes to the pathogenesis of morbidity in the cardiovascular, pulmonary, genitourinary, and gastrointestinal systems. In the presence of dysregulation of the ANS, receptors of the parasympathetic (PNS) and sympathetic (SNS) branches are targeted for heart rate dysrhythmias, blood pressure dysregulation, or airway hyperreactivity. In man, there is a paucity of evidence on the role of the ANS and, more specifically, the SNS regulation of hepatic synthesis and secretion of triglyceride-rich lipoproteins (TRL) in man. The liver is innervated from SNS postganglionic neurons that arise from preganglionic projections originating from the 5^th^ through 12^th^ thoracic vertebrae to the celiac and superior mesenteric ganglion [1,2]. Somewhat conflicting preclinical evidence exists as to how SNS modulation influences the synthesis of TRL products and on the serum lipid profile [3–8]. In preclinical models of sympathoexcitation through manipulation of the α_1_ adrenoceptor, serum very low density lipoproteins (VLDL), apolipoprotein B (ApoB), and triglyceride (TG) concentrations were demonstrated to decline [3,4,6], while in another report [8], antagonism of the α_1_-adrenoceptor increased lipoprotein lipase (LPL) activity and depressed hepatic TG synthesis and secretion. Conversely, in sympathodenervation, a lower rate of fatty acid oxidation and incorporation into the VLDL was associated with an overall decline in VLDL secretion [5,7]. Thus, the question remains as to the effect of functional SNS denervation on hepatocyte fatty acid incorporation into VLDL and hepatic secretion of VLDL.

Activation of the SNS results in adipocyte lipolysis via stimulation by norepinephrine through protein kinase A-mediated phosphorylation of hormone-sensitive lipase and perilipin [9]. Catecholamine-induced lipolysis is relatively increased in the visceral adipose tissue (VAT) depot possibly, in part, as a consequence of functional changes in adrenoreceptor subtypes related to increased β_3_-adrenoreceptors and decreased α_2_-adrenoceptor function [10,11]. Lipolysis and fatty acid turnover has been observed to be higher in visceral adipocytes than in subcutaneous adipocytes, and the visceral adipocytes have been shown to be less responsive to the anti-lipolytic effect of insulin [12]. The increased release of free fatty acids (FFA) into the portal circulation induces adverse changes in insulin signaling and increases hepatic insulin resistance [13]. As such, sympathetic activity to the abdominal VAT, especially if increased, would be assumed to be associated with increased release of FFA into the portal circulation, resulting in hepatic storage of fatty acids or release of TRL particles [10]; decreased sympathetic activity to visceral adiposity resulting from any etiology would be anticipated to have a contrary effect. Thus, it may then be speculated that the loss of functional SNS innervation to the abdomen due to spinal cord injury (SCI) would result in a reduced circulating number and concentration of TRL for any given degree of insulin resistance and adiposity.

SCI results in a complete or partial interruption of sensorimotor control below the neurological level of injury, and a functional disruption or complete ablation of SNS modulation of sublesional visceral function. Guidelines exist to classify residual sensory and motor function below the level of injury [14], but classification of residual ANS function has not yet been developed for the degree of specific end-organ function [15]. Paralysis to the lower extremities results in severe muscle atrophy, markedly reduced physical activity, and often increased visceral adiposity, insulin resistance and associated impaired glucose tolerance or diabetes mellitus [16–23]. If findings of reduced TRL production after sympathodenervation from experimental animal models are transferable to man, it would be appropriate to hypothesize that persons with SCI, whose lesion is proximal to the thoracic projections of the celiac and superior mesenteric ganglia, and thus to the liver and abdominal VAT, would present with reduced serum VLDL concentrations compared to persons with SCI whose injury is distal to or including the spinal levels having hepatic SNS projections.

## Methods

### Subjects

One hundred-three men with non-ambulatory SCI [i.e., American Spinal Injury Association Impairment Scale (AIS) designation of A, B, or C: A = complete loss of sublesional motor and sensory function; B = complete loss of sublesional motor function and incomplete loss of sublesional sensory function; C = incomplete loss of sublesional motor and sensory function with the majority of muscles having less than functional strength] [14], chronic SCI (>12 months post-SCI) were recruited for participation from the Spinal Cord Injury Service and outpatient SCI clinics of the James J. Peters Veterans Affairs Medical Center (JJP VAMC), Bronx, NY, and outpatient clinics at the Kessler Institute for Rehabilitation, West Orange, NJ; 53 able-bodied (AB) men were recruited from outpatient clinics and hospital staff at the JJP VAMC, Bronx, NY. Men with or without SCI between the ages 20 and 65 were considered eligible for study if they were free from acute medical illness (i.e., not receiving treatment for an active medical condition), without diagnosed chronic illness (i.e., heart disease, pulmonary disease, diabetes mellitus), and had the capacity to provide informed consent. No subjects were taking medications with known effects on any of the study-related outcome measurements; this list may include, but was not limited to all classes of hypolipidemic agents, insulin or peripheral insulin-sensitizing agents, hormone-replacement therapies, sympathomimetics or their antagonists. The study protocol was approved by the Institutional Review Board of the James J. Peters VA Medical Center and the Kessler Institute for Rehabilitation. Written informed consent or verbal assent (for those with impaired hand function) was obtained from each subject prior to study participation.

### Methods

Subject data was collected during a single study visit. All participants were required to complete an overnight fast of at least 12 hours prior to arriving for study evaluation at the testing center between 8 and 11 a.m., which included a brief medical history and seated blood pressure measurement, venous blood collection for determination of the lipid profile, lipid particle concentration and size, glucose and plasma insulin concentrations, and a dual energy x-ray absorptiometry (DXA) scan. Venous blood samples were analyzed for the serum lipid profile [total cholesterol, TG, HDL-C and estimated LDL-C] in the General Chemistry Laboratory of using an ADVIA 1650 automatic chemistry analyzer following standard procedures recommended by the manufacturer (Bayer Diagnostics, Newbury, UK). Apolipoprotein A1 and B concentrations were quantified by a commercial laboratory (Quest Diagnostics, Teterboro, NJ) using standard commercial procedures. The concentration and size of lipid particles were determined using automated NMR Lipoprofile 3 (LP3) lipoprotein particle analysis, by previously described methods (LabCorp, Raleigh, NC) [24,25]. From the LP3 technique, lipid particle (P) concentrations were determined for Total VLDL (VLDL-P) and its respective Large and Small subparticle fractions. Fasting plasma insulin (FPI) samples were batch processed in duplicate by radioimmunoassay [26]. Fasting plasma glucose (FPG) concentrations were performed on an automated glucose analyzer (YSI 2300 STAT Plus, YSI Life Sciences, Yellow Springs, OH). From the FPG and FPI concentrations, homeostatic model assessment of insulin resistance (HOMA-IR, model 2) was calculated [27] using software available on the internet (www.OCDEM.ox.ac.uk). Individual information from the medical history, intake exam and total cholesterol and HDL-C concentrations were used to calculate the 10 year Framingham Risk Score (http://cvdrisk.nhlbi.nih.gov/) [28], which was used as a covariate in our statistical models.

A total body scan was performed in accordance with the manufacturer’s guidelines using a fan beam DXA machine (GE Healthcare Lunar iDXA, platform version 13.6, GE Healthcare Lunar, Madison, Wisconsin, USA). Proprietary software algorithms from the manufacturer were used to analyze the scans and compute total body fat mass and standard regions of interest (ROI) for each participant. From a DXA total body image, subcutaneous adipose tissue (SAT) mass and VAT mass were obtained from the abdominal android fat (AF) mass region by using iDXA enCoreTM CoreScan software package, which is a FDA-approved software upgrade for the iDXA that has been validated against computer tomography (CT; R^2^ = 0.957) to measure VAT [29]; a recent report from our group demonstrated the utility of this measure to capture abdominal adiposity in persons with SCI [30]. The abdominal AF mass ROI was defined as the area that begins at the proximal border of the iliac crest and has a height that is 20% of the total distance from the proximal border of the iliac crest to the base of the skull. To measure SAT, the x-ray attenuation of the abdominal cavity in the android region was obtained using the width of the SAT layer on the lateral aspects of the abdomen, the anterior-posterior thickness of the abdomen, and geometric assumptions of fat distribution. The mass for VAT was calculated by subtracting the SAT from total AF. Fat mass (grams) for the SAT, VAT, and AF regions were transformed to volumes using a constant correction factor (density of adipose tissue = 0.94 g/cm^3^) and reported as SAT volume, VAT volume, and AF volume. After completing the total body DXA scan, participants remained in the supine position and waist circumference was measured with a flexible measuring tape at the midpoint between the proximal border of the iliac crest and the lower margin of the last palpable rib in the mid-axillary line at the end of several consecutive natural breaths [31]. All measures were taken in duplicate to the neatest 0.5 cm and the mean value was reported.

### Statistics

For statistical comparison, the SCI cohort was dichotomized by group for the neurological level of injury. Subjects with SCI at or proximal to the 4^th^ thoracic vertebrae (↑T4) were assumed to have partial or complete loss of supraspinal-mediated sympathetic hepatic function and VAT innervation, whereas subjects with SCI that was at or distal to the 5^th^ thoracic vertebrae (↓T5) were assumed to have supraspinal-mediated sympathetic function to abdominal tissues. Separate factorial analysis of variance (ANOVA) were performed to identify the presence of group differences in subject demographic, anthropometric, body composition, systolic blood pressure, 10-year Framingham risk for a cardiac event, HOMA-IR and FPG, FPI and fasting lipid profiles. Pearson chi-square tests were performed to determine if the groups differed in the number of smokers, and for the SCI cohort only, the distribution of AIS designation between sub-groups.

To accommodate the presence of significant group differences among select demographic, body composition, fasting serum/plasma profiles, and computed variables with an appreciated association and/or mechanistic role or influence on the synthesis of the primary study outcome measurements (i.e., TG, Total VLDL-P, Large VLDL-P and Small VLDL-P), separate multiple regression analyses were performed to identify variables that provided a significant contribution to the prediction of the lipoprotein and lipid particle concentrations of interest. Once significant independent variables were identified from the respective multiple regression analyses, these served as covariates in separate factorial analyses of covariance (ANCOVA) that were performed to identify statistically significant differences between groups; the resulting estimated marginal means and 95% confidence intervals (CI) were recorded and are presented as “adjusted” outcome measures. The following variables were used as covariates in their respective statistical model: TG (10-year Framingham Risk score, VAT volume); Total VLDL-P (age, 10-year Framingham Risk score); Large VLDL-P (10-year Framingham Risk score); and Small VLDL-P (age, 10-year Framingham Risk score).

To demonstrate the potential presence of linearity between the primary study outcome measurements (i.e., unadjusted TG, Total VLDL-P, Large VLDL-P and Small VLDL-P) and HOMA-IR and VAT volume, simple regression analyses were performed for each group. For those relationships where linear significance was identified, separate F-tests were performed to determine if the respective slope of the regression line significantly deviated from zero; ANCOVA were then performed to determine if the respective slopes (relationship of TRL measurement to HOMA-IR or VAT) were different between groups with HOMA-IR or VAT volume serving as the respective covariate in each model. Individual subject data were plotted per the group designations and the slopes presented for those linear models that were identified as being significant (performed with GraphPad Prism version 5.04 for Windows, GraphPad Software, San Diego, CA, USA). All other statistical analyses were completed using IBM SPSS Statistics 21 (IBM, Armonk, NY, USA). An *a priori* level of significance was set at p≤0.05.

## Results

Demographic, anthropometric, and other descriptive characteristics for each of the three study groups are provided (Table 1). The groups were not significantly different for body weight, AF and SAT volume, the number of smokers, or DOI. The proportion of subjects with AIS A, B, and C classifications was not significantly different among the groups, with 64, 15, and 21%, respectively, in the ↓T5 group and 53, 18, and 29% for AIS classifications A, B, and C, respectively, in the ↑T4 group. The AB group was younger, had a smaller waist circumference, less total body fat, and a lower VAT volume compared to the two SCI groups, which were quite similar in VAT volume. Because of differences in height, the BMI was significantly lower in the ↑T4 group compared to the AB group. As expected because of cardiovascular sympathetic dysfunction, the ↑T4 group also had a significantly lower systolic BP compared to the AB and ↓T5 groups. The SCI groups did not differ for DOI or the proportion of subjects by AIS impairment classification (Table 1).

**Table 1:** Characteristics of study cohorts.

	AB	↓ T5	↑ T4	p-value	Post-Hoc
n	53	48	55	-	
Age (yrs)	40.1 (11.2)	48.4 (13.0)	45.7 (11.1)	<0.01	1,2
Height (m)	1.74 (0.08)	1.77 (0.07)	1.79 (0.08)	<0.01	2
Weight (kg)	85.9 (18.8)	88.1 (19.2)	83.2 (19.8)	NS	
BMI (kg/m^2^)	28.3 (5.2)	27.9 (4.9)	25.8 (4.9)	<0.05	2
Waist Circumference (cm)	92.2 (13.7)	100.7 (14.1)	100.0 (16.5)	<0.01	1,2
Total Body Fat (kg)	24.5 (12.7)	32.6 (11.6)	30.7 (12.6)	<0.01	1, 2
Android Fat Volume (cm^3^)	2343 (229)	2866 (240)	2924 (229)	NS	
Subcutaneous Adipose Tissue Volume (cm^3^)	1247 (184)	862 (1100)	929 (992)	NS	
Visceral Adipose Tissue Volume (cm^3^)	1107 (969)	2101 (1155)	1983 (1301)	<0.0001	1, 2
AIS A / B / C (n)	-	31 / 7 / 10	29 / 10 / 16	NS	
DOI (yrs)	-	15.4 (11.0)	20.0 (12.7)	NS	
Smokers (n)	7	7	3	NS	
Systolic Blood Pressure (mmHg)	118 (8)	122 (26)	98 (13)	<0.0001	1, 3
10-Year Framingham Risk (%)	4.2 (2.5)	7.4 (6.1)	4.9 (3.6)	<0.001	1, 2

Data are expressed as group mean (SD). AB = able-bodied; BMI = body mass index; AIS = American Spinal Injury Association Impairment Scale; DOI = duration of injury. P-values represent significant group main effects. Significant comparisons from post-hoc analyses: ^1^AB v. ↓T5 = p<0.05; ^2^AB v. ↑ T4 = p<0.05; ^3^↓T5 v. ↑ T4 = p<0.05

Fasting concentrations of blood and lipid markers are provided (Table 2). There were significant differences among the groups for FPG and FPI, but the mean values for the groups were within the normal range. The mean HOMA-IR scores for the groups trended towards statistical difference, but each mean value remained in the normal range; 8 (15%), 12 (25%) and 13 participants (24%) in the AB, ↑T4, and ↓T5 groups, respectively, were classified as being insulin resistant (HOMA-IR ≥1.85) [32]; as such, the number of participants in the two SCI groups who were insulin resistant were comparable and did not differ statistically. The ↑T4 group had the lowest mean concentration of serum total cholesterol compared to other groups, a comparison which approached significance (p = 0.06). The serum mean TG concentration was significantly higher in ↓T5 group compared to the ↑T4 group and AB groups (Table 2). The SCI cohorts had significantly lower mean serum HDL-C and apolipoprotein A1 concentrations compared to that in the AB group (Table 2). The mean serum ApoB concentration was significantly higher in the ↓T5 group than that in the ↑T4 group. Lipid NMR spectroscopy revealed that the mean Total VLDL-P concentration was significantly greater in the ↓T5 group than that in the two other groups, partially explaining the higher mean ApoB concentration observed in the lower cord lesion group, which may also be explained to a degree by a relatively, but not significantly, higher mean LDL-C concentration in the↓T5 group than that in the ↑T4 group. Subclass analyses of the VLDL-P revealed that the ↓T5 group had a significantly higher mean Large VLDL-P concentration than that in the AB group, and the ↓T5 group had a higher mean Small VLDL-P concentration than that in the ↑T4 group (Table 2).

**Table 2:** Unadjusted fasting blood and lipid profiles by group.

	AB	↓T5	↑ T4	p-value	Post-Hoc
Glucose (mmol/l)	5.0 (4.7, 5.2)	4.7 (4.5, 5.0)	4.3 (4.1, 4.6)	<0.001	1
Insulin (mU/ml)	8.3 (5.9, 10.8)	12.5 (9.9, 15.0)	11.8 (9.3, 14.2)	<0.05	
HOMA-IR	1.09 (0.80, 1.39)	1.54 (1.23, 1.86)	1.47 (1.18, 1.77)	0.08	
Total Cholesterol (mg/dl)	183 (172, 194)	185 (174, 197)	167 (156, 178)	0.06	
Triglycerides (mg/dl)	101 (83, 119)	150 (131, 168)	112 (95, 130)	<0.001	1, 2
HDL-C (mg/dl)	50 (48, 53)	42 (39, 45)	42 (39, 45)	<0.0001	1, 3
LDL-C (mg/dl)	112 (103, 120)	113 (104, 122)	102 (93, 111)	NS	
Apolipoprotein A1 (mg/dl)	143 (137, (149)	127 (120, 133)	122 (112, 125)	<0.0001	1, 3
Apolipoprotein B (mg/dl)	87 (80, 94)	99 (91, 106)	82 (75, 89)	<0.01	2
Total VLDL-P (μmol/l)	58.0 (50.1, 65.8)	74.3 (66.1, 82.6)	55.1 (47.7, 62.8)	<0.01	2, 3
Large VLDL-P (μmol/l)	3.4 (2.1, 4.6)	6.0 (4.6, 7.4)	4.5 (3.2, 5.8)	<0.05	3
Small VLDL-P (μmol/l)	32.1 (27.4, 36.8)	39.6 (34.6, 45.5)	30.8 (26.1, 35.5)	<0.05	2
VLDL-P Size (nm)	46 (44, 48)	50 (48, 52)	49 (47, 51)	<0.05	

Data are expressed as group mean (95% CI). AB = able-bodied; HOMA-IR = homeostatic model assessment of insulin resistance; NMR = nuclear magnetic resonance; VLDL: very low density lipoprotein; P = particle. P-values represent significant group main effects. Significant comparisons from post-hoc analyses: ^1^AB v. ↑T4 = p<0.05; ^2^↑T4 v. ↓T5 = p<0.05; ^3^AB v. ↓T5 = p<0.05.

**Fig 1:**
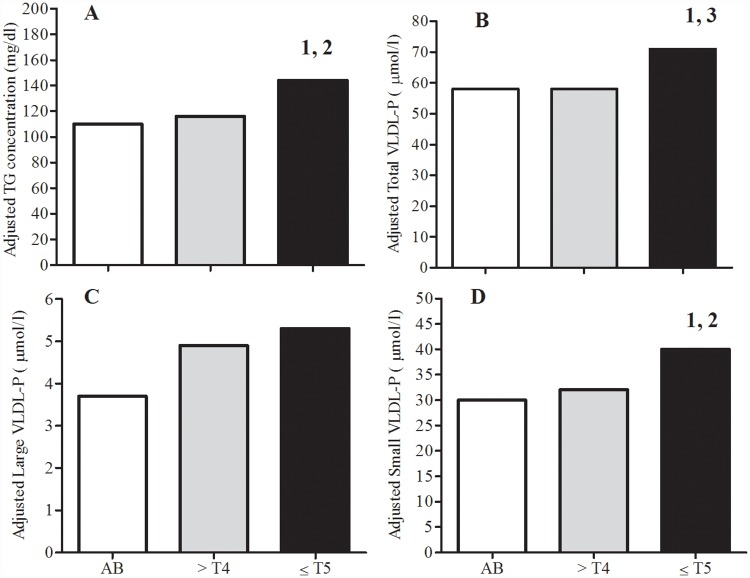
Distribution of adjusted triglyceride-rich lipoproteins by group. Data are presented as estimated marginal means by group. 95% CI for groups (i.e., AB, ↑T4, ↓T5) are provided for Adjusted TG (A: 94, 127; 100, 132; 126, 162), Adjusted Total VLDL-P (B: 50, 65; 51, 65; 63, 79), Adjusted Large VLDL-P (C: 2.4, 4.9; 3.7, 6.1; 4.0, 6.5), and Adjusted Small VLDL-P (C: 25, 35; 27, 36; 35, 45), respectively. Significant Bonferoni post-hoc test comparisons for group differences: ^1^p<0.05, ↑T4 vs. ↓T5; ^2^p = 0.07, AB vs. ↓T5; ^3^p<0.05, AB vs. ↓T5.

Because the three groups had statistical differences for demographic, anthropometric and other descriptive variables that may have influenced the synthesis and concentration of circulating TRL, multiple regression analyses were performed and the statistically adjusted concentrations are reported as the estimated marginal means by group and lipoprotein (Fig 1A–1D). The adjusted mean serum TG concentration (Fig 1A) and Total VLDL-P (Fig 1B) and Small VLDL-P concentrations (Fig 1D) were significantly higher in the ↓T5 group than that in the ↑T4 group. The adjusted mean Total VLDL-P concentration was significantly higher in the ↓T5 group than that in the AB group (Fig 1B), but the adjusted mean serum TG concentration (Fig 1A) and Small VLDL-P concentration (Fig 1D) were also higher in the ↓T5 group but only approached statistical significance compared to the AB group (p = 0.07). The previously described significant difference between the ↓T5 and AB groups for unadjusted mean Large VLDL-P concentration (Table 2) did not retain significance after applying the analyses with covariates (Fig 1C).

**Fig 2:**
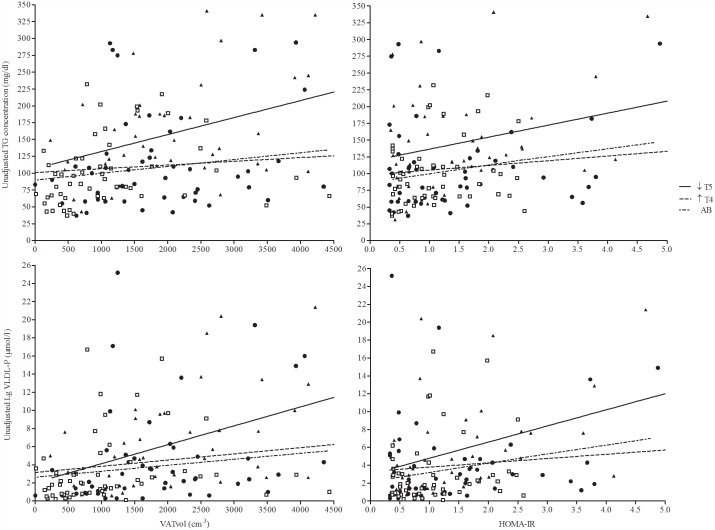
Relationships between HOMA-IR and VAT volume and unadjusted TG concentration and number of large VLDL-P, respectively by group. In the ↓T5 group, the slope of the regression line significantly deviated from zero (p<0.05) in the respective relationships between unadjusted TG concentration and VAT volume (A) and HOMA-IR (B) and, for unadjusted Lg VLDL-P and VAT volume (C) and HOMA-IR (D), but not for the other groups. Similarly, the slope of the regression line for the ↓T5 group was significantly different (p<0.05) than the other groups for both unadjusted TG concentration models (A, B). Regression equations:
(A)AB: TG = 89.81+0.01(VATvol)↑T4: TG = 101.10+0.006(VATvol)↓T5: TG = 107.02+0.25(VATvol); r^2^: 0.15, p<0.05(B)AB: TG = 86.33+13.31(HOMA-IR)↑T4: TG = 98.14+7.01(HOMA-IR)↓T5: TG = 118.62+19.92(HOMA-IR); r^2^: 0.10, p<0.05(C)AB: LgVLDL-P = 2.62+0.001(VATvol)↑T4: LgVLDL-P = 3.15+0.001(VATvol)↓T5: LgVLDL-P = 2.04+0.002(VATvol); r^2^: 0.22, p<0.05(D)AB: LgVLDL-P = 2.15+1.04(HOMA-IR)↑T4: LgVLDL-P = 3.32+0.48(HOMA-IR)↓T5: LgVLDL-P = 2.99+1.81(HOMA-IR); r^2^: 0.20, p<0.05

To determine the presence of linearity between the TG concentrations or Large VLDL-P concentration and HOMA-IR or VAT volume, separate simple regression analyses were performed (Fig 2). In the ↓T5 group, the slope of the regression line significantly deviated from zero (p<0.05) in the relationship between unadjusted serum TG concentration and VAT volume (Fig 2A) or HOMA-IR (Fig 2B) and for Large VLDL-P concentration and VAT volume (Fig 2C) or HOMA-IR (Fig 2D). The ↑T4 and AB groups did not display significant relationships between serum TG or Large VLDL-P concentration with VAT volume or HOMA-IR (Fig 2A–2D). Therefore, individuals with SCI who had intact sympathetic hepatic function with higher VAT and IR were more likely to have higher concentrations of serum TG and Small VLDL-P than were those with comparable predisposing conditions but lacking functional sympathetic innervation to the abdomen.

## Discussion

Our report is the first to demonstrate in humans that loss of functional sympathetic innervation to the liver and VAT appears to favorably impact circulating TG concentrations and concentrations of total and specific subclasses of VLDL-P. The ↑T4 group, that is presumed to have a loss of functional innervation to the liver and VAT, had significantly lower average serum TG, Total VLDL-P, and Small LDL-P concentrations than that observed in the ↓T5 group, with the latter group of lower level lesions presumed to have relatively normal physiological SNS function of the abdominal tissues. The significance of these findings persisted even after controlling for demographic differences between the groups. It is also worth emphasizing that the ↑T4 and ↓T5 groups had similar VAT volume and proportion of participants with insulin resistance. Of note, despite having a greater VAT volume than the AB group, the ↑T4 group had a mean serum TG and VLDL-P concentrations that were not significantly differ than that of the AB group. The SCI group with preserved hepatic SNS modulation (e.g., ↓T5 group) had a TRL profile that was more adverse than the AB group presumably, at least in part, as a consequence of a larger adipose burden and relatively increased insulin resistance. When both VAT volume and insulin resistance were controlled for in the statistical analyses, as well as other factors that may have influenced relationships of interest with the serum lipid parameters, the major unadjusted lipid findings were supported. Thus, one could argue that persons with SCI at or distal to T5 who are overweight/obese may warrant more aggressive clinical monitoring and, if indicated, initiation of pharmacological or rehabilitation intervention(s) to increase the level of activity and, if possible, reduce adiposity in an effort to maintain peripheral insulin sensitivity and thus improve the lipid profile and associated risk of cardiovascular disease.

Insulin resistance can contribute to a complex sequelae of metabolic processes that alter molecular signaling in and function of the hepatocyte [33], skeletal muscle, and adipocyte [34]. Excess glucose that is not stored as glycogen may be degraded to three carbon fragments that are converted to TGs by the liver. *De novo* synthesized TG is utilized for heat production via ATP generation or is stored in white adipose tissue (WAT) as a depot for future lipolysis [35,36]. The insulin resistant state may have an opposing effect to that of β-adrenergic stimulation at the level of the adipocyte. SNS activity is increased in human obesity [37], which may be, in part, related to a state of hyperinsulinism [38], although controversy exists because obese individuals do not appear to retain sensitivity to the stimulatory effects of insulin on the SNS [39]. TG stored in WAT is mobilized and broken down into glycerol and FFA during lipolysis in response to β-adrenergic stimulation [40]. The impairment of adipocyte β-adrenergic responses to stimulation by the SNS, or as a consequence of insulin resistance, may be associated with metabolic disorders that lead to a reduced expression of genes that are critical regulators of glucose and lipid metabolism [41]. However, it should be appreciated that β-blocker administration in man usually results in increased serum TGs and decreased serum HDL-C levels [42], while selective α_1_-adrenoceptor inhibitors result in stimulation of LPL activity and reduced VLDL synthesis and secretion [8], which would serve to reduce the level of circulating TGs. The overproduction of VLDL by the liver is a cardinal feature of insulin resistance, due to reduced inhibition of VLDL secretion [43]. In addition, insulin deficiency states are associated with depressed LPL activity [44,45], reducing the clearance of TGs and, as such, increased levels of serum VLDL and associated remnant particles.

In our participants with higher cord lesions, the loss/reduction of sympathoexcitation to the abdominal fat depot, and therefore diminished lipolysis, may be speculated to have been a contributing factor for the accumulation of visceral adiposity. Those with higher cord lesions are also generally less active, which may predispose to abdominal obesity as well. SAT volume, as expected, was similar among all groups [46], as was AF volume, but VAT volume in each of the SCI groups was similar and about two-fold higher than that observed in the AB group, confirming previous work related to visceral adiposity in those with SCI despite similar body mass index values to able-bodied cohorts [30]. Accumulation of VAT is strongly associated with the metabolic syndrome, type 2 diabetes mellitus, and heightened risk of cardiovascular disease [47,48], and visceral adiposity is positively associated with insulin resistance [49]. These morbid conditions are more highly prevalent in the SCI than the AB population [16–23,50–54]. The anti-lipolytic effect of elevated circulating insulin levels in insulin resistant states will still serve to inhibit lipolysis, an effect which may be accentuated in the absence of functional sympathetic innervation; in addition, the spillover of epinephrine from sympathoexcitation of the adrenal medulla in those with higher cord injuries would be assumed to be dramatically blunted [55–57]. An expanded VAT volume has been reported by several groups to result in a population of large, insulin resistant adipocytes with macrophage infiltration and an associated increased expression of inflammatory mediators that may serve to further aggravate the insulin resistant state [58–60].

Serum HDL-C concentrations in persons with SCI are often much lower than an appropriately matched AB group [61]. Of note, when the lipid profile is investigated in a sufficient number of persons with SCI, the group with higher spinal cord lesions has been observed to have a more depressed mean serum HDL-C concentration than that of lower cord lesions [21], which has been attributed to the dramatically reduced level of physical activity and a greater average degree of insulin resistance in those with tetraplegia; such a relationship for serum HDL-C concentration between groups with higher and lower spinal cord lesions was not anticipated to be observed in our relatively small cohort because of the appreciated individual genetic variability. An inverse correlation between serum TGs and HDL-C levels has been well appreciated in the general population, and such a relationship has also been described in those with SCI [21,22,62]. Circulating TGs can deplete cholesterol in HDL-C by a two-step process by which TG replaces cholesterol ester one-to-one in the lipid core through the action of cholesterol ester transfer protein, and the incorporated TG in the HDL-C is then hydrolyzed by the action of lipases, which ultimately results in cholesterol-depleted HDL-P of reduced size [63] that is more readily metabolized and/or excreted by the kidney [64,65]. Thus, it would be expected from an appreciation of this lipid degradation pathway that those with higher serum TG concentrations should also have lower serum HDL-C levels; however, this assumption does not appear to hold true in those with higher spinal cord lesions [21]. Another possibility that has not been considered to date, but should probably be raised in light of the observations with regard to circulating TRLs in this report, is that impairment of SNS function to the liver per se may be partially responsible for lower circulating HDL-C levels in persons with higher cord lesions.

## Conclusions

The loss of functional SNS hepatic innervation resulted in a relative reduction of circulating TRL products in persons with SCI lesions proximal to and including T4 compared to those with an injury at or distal to T5. The lower level of serum TRL products in the group with higher cord lesions was apparent despite elevated, but comparable, levels of VAT in the group with the lower cord lesions and the proportion of participants in both SCI groups with insulin resistance. In contrast, preservation of functional SNS hepatic innervation in persons with lower spinal cord lesions with enlarged VAT compartment may be assumed to have contributed to the observed relative elevation of serum TRL products compared to that in AB subjects. As such, a loss of normal sympathetic innervation to the abdomen appears to impart a mixed cardio-protective pattern upon serum lipids, with an observed reduction in serum TRL products, which is assumed to be beneficial, but also associated with a depressed serum HDL-C concentration. Our findings suggest a new paradigm in which functional sympathetic innervation to the abdomen should be recognized as an important contributing factor in the prediction of the concentration of circulating TRL products, which may play a role in the genesis and perpetuation of cardiometabolic dysfunction and atherosclerotic disease.
